# A 24-Year-Old Male Patient With an Ostium Secundum Complex Atrial Defect Secondary to a Perforated Aneurysm With Inferior Vena Cava Agenesis

**DOI:** 10.7759/cureus.61624

**Published:** 2024-06-03

**Authors:** Atl Simon Arias Rivera, Katherine Rubio, Lizbeth S Gonzalez Solano, Mauricio Damian Gomez Gonzalez, Alain Ledu Lara, Manuel Carrillo Cornejo, Moises C Calderon Abbo

**Affiliations:** 1 General Surgery, Hospital Angeles Lomas, Huixquilucan, MEX; 2 Surgery, Hospital Angeles Lomas, Huixquilucan, MEX; 3 Surgery, Universidad Nacional Autonoma de Mexico, Mexico City, MEX; 4 Cardiac Surgery, Hospital Angeles Lomas, Huixquilucan, MEX; 5 Cardiothoracic Surgery, Hospital Angeles Lomas, Huixquilucan, MEX; 6 Interventional Cardiology, Hospital Angeles Lomas, Huixquilucan, MEX

**Keywords:** echocardiogram, congenital heart disease, agenesia, inferior vena cava, surgery, atrial septal defect

## Abstract

The article describes a successful clinical outcome in the case of a 24-year-old male with a diagnosis of an ostium secundum atrial defect secondary to a perforated aneurysm associated with vena cava agenesis. During hospitalization, an echocardiogram revealed the presence of ostium secundum inter-atrial communication with a left to right shunt, a left ventricular ejection fraction (LVEF) of 60%, and mild pulmonary hypertension, measured at 40 mmHg. CT imaging showed anomalous dilation of the azygos vein (16.8 mm), associated with interruption of the vena cava in the intrahepatic and adrenal portion, continuing through the azygos system and draining into the superior vena cava. Open-heart surgery was performed with pericardium patch placement on the defect. Postoperative transthoracic echocardiography revealed a tracking of the interatrial septum, with adequate placement of the surgical patch and no evidence of residual short circuits. The postoperative recovery was favorable, and the patient was discharged five days after surgery. Outpatient monitoring at the first and third months showed no complications during physical examination and echocardiogram imaging.

## Introduction

Congenital heart disease (CHD) encompasses structural abnormalities of the heart that are present at birth and arise in the fetus during embryonic development in the uterus. Approximately half a million adults in the United States (US) are affected with congenital cardiac disease and approximately 1% of children are born with congenital cardiac problems as a result of genetic or chromosomal disorders [1].

Although CHD is mostly diagnosed during the neonatal period, or early childhood, diagnosis of these conditions during adulthood isn’t unheard of. Atrial septal defect (ASD) is the primary type of CHD that is first diagnosed in adulthood. The estimated prevalence of ASDs in adults is 0.88 per 1000 patients [2].

The term ASD includes a group of abnormalities that allow communication between left and right atria. The location of the defect categorizes ASD into five types: patent foramen ovale (PFO), ostium secundum defect (OSD), ostium primum defect (OPD), sinus venosus defect (SVD), and coronary sinus defect (CSD) [3].

Secundum atrial septal defect refers to the communication within the fossa ovalis. It is considered the most prevalent cardiac abnormality present at birth that continues into adulthood (80% of cases). It is responsible for around 30% of newly diagnosed cases. It occurs due to a lack of tissue near the fossa ovalis [3].

OPD refers to the initial opening or entrance. In comparison to OSD, SVD anomalies are more frequently observed in the superior portion of the embryologic sinus venosus and are associated with partial anomalous pulmonary venous return. CSD is a rare form of ASD that is typically difficult to diagnose. However, an exposed coronary sinus can be seen when agitated saline contrast bubbles originating from the left upper extremities enter the left atrium (LA) prior to the right atrium (RA) [3-4].

Frequently the history of the patient uncovers a progressive alteration in exercise capacity, typically described as modest, and sometimes obvious difficulty in breathing during physical effort. While ASD is typically characterized by a gradual clinical progression and lack of symptoms in patients, its detection helps prevent time-related consequences, including arrhythmias, thrombosis, right heart failure, and pulmonary arterial hypertension (PAH) [5].

We present a case of a 24-year-old male patient with a large interatrial defect secondary to a perforated aneurysm presented with inferior vena cava (IVC) agenesis.

## Case presentation

**Figure 1:**
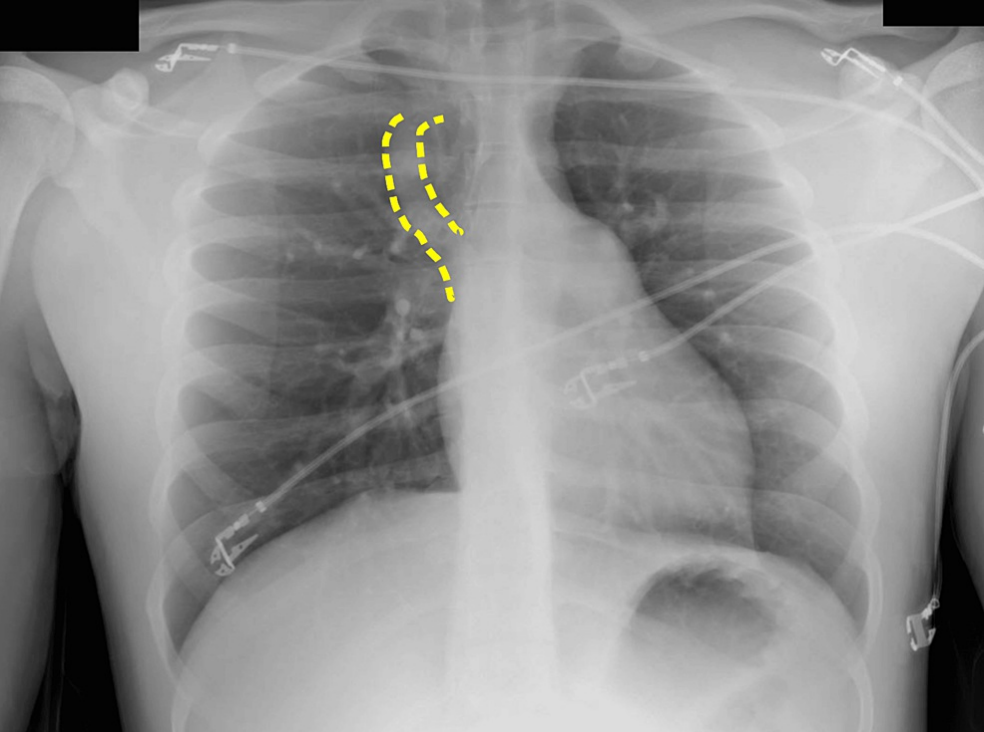
Posterior anterior (PA) chest X-ray Aorta with normal course and density. Image suggesting prominence of the azygos vein (yellow intermittent line). Both pulmonary hilia without evidence of lesions. The cardiac silhouette preserves shape, size, and contours.

**Figure 2:**
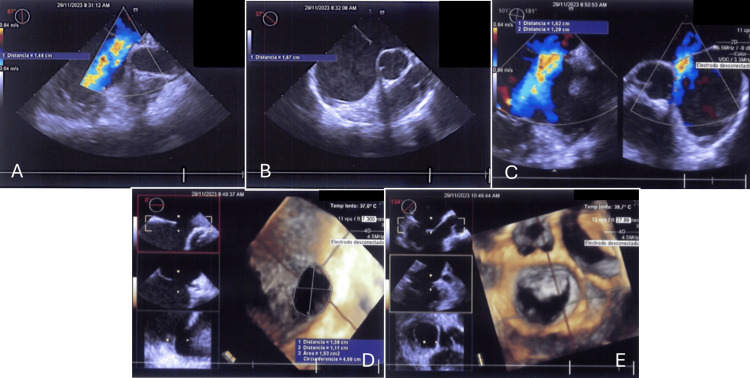
Preoperative transthoracic echocardiogram A-C: Ostium secundum-type atrial septal defect, with shunts from left to right, with agenesis of the inferior vena cava. D-E: Inter-atrial septum with a defect of 1.5 cm x 1.2 cm, with shunt from left to right, with a posterior edge of 1.6 cm, an upper edge of 0.6 cm, an inferior edge of 1.1 cm, and a short aortic edge of 4 mm.

**Figure 3:**
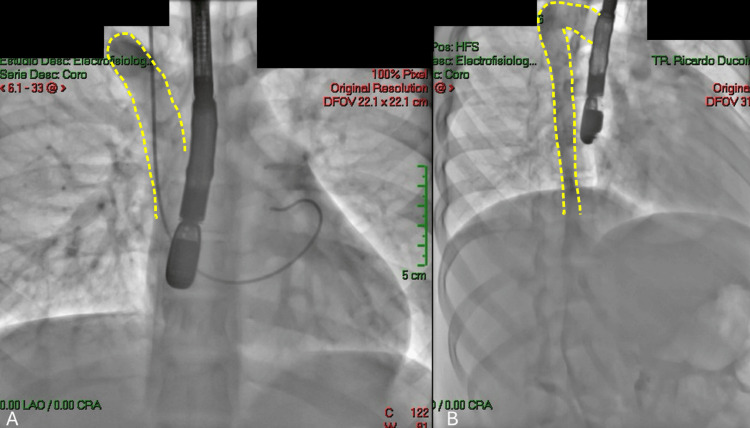
Catheterization performed via the internal jugular vein A-B: Presence of a prominent azygos vein (yellow intermittent line) with a catheter line through a jugular internal vein approach localized in the right atrium.

**Figure 4:**
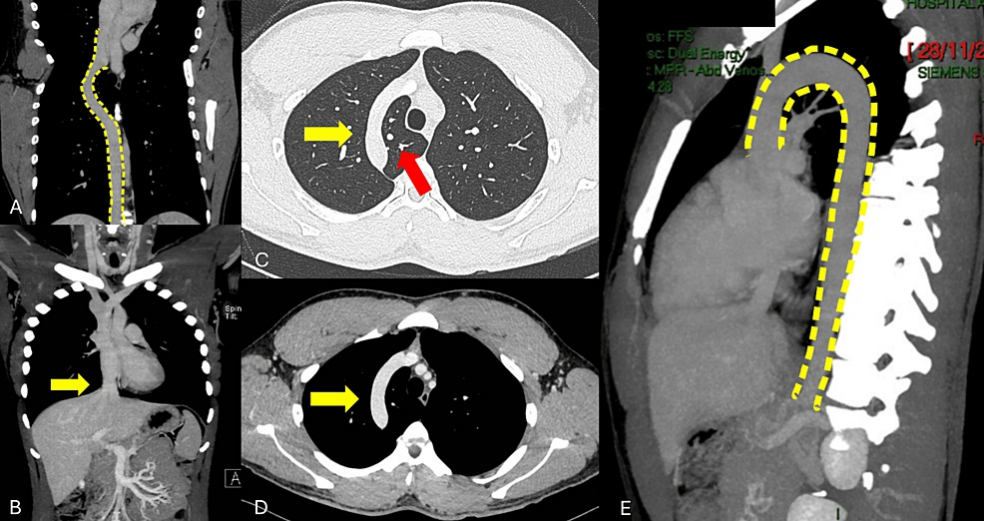
Contrasted thoracoabdominal computed tomography A-B: Anomalous dilation of the azygos vein (16.8 mm) (intermittent yellow line), associated with interruption of the inferior vena cava in the intrahepatic and adrenal portion (yellow arrow). C-D: Presence of dilation of the azygos vein (yellow arrow) in a transversal plane with the presence of an azygos pulmonary lobe (red arrow). E: Continuation through the azygos system (intermittent yellow line) and drainage of the superior vena cava.

A 24-year-old male patient presented with two months of evolution with progressive dyspnea and multiple episodes of orthopnea at night. The symptoms progressed with daily episodes accompanied by chest palpitations and precordial pain exacerbated with physical activities. Upon arrival at the emergency department, the physical exploration showed a wide, fixed splitting of S2. The electrocardiogram sinus rhythm without enlargement of cardiac cavities. The diagnostic evaluation began with an echocardiogram and a chest X-ray that showed images suggesting the prominence of the azygos vein (Figure 1). The transthoracic echocardiogram showed the presence of ostium secundum interatrial communication of 1.5 cm x 1.2 cm with a left to right shunt from a perforated atrial aneurysm, a left ventricular ejection fraction (LVEF) of 60%, and mild pulmonary hypertension of 40 mmHg (Figure 2). The patient was programmed for a percutaneous closure intervention with an Amplatzer device, which failed because of a vascular variant with evidence of inferior vena cava agenesis (Figure 3). Subsequent to these findings, a contrasted thoracoabdominal computed tomography (CT) was performed. The CT showed an anomalous dilation of the azygos vein (16.8 mm), associated with interruption of the inferior vena cava in the intrahepatic and adrenal portion, with continuation through the azygos system and drainage of the superior vena cava. The suprahepatic veins drain to the right atrium with the rest of the study with no abnormalities (Figure 4). Based on these findings, the patient was scheduled for open-heart surgery. 

A supine position was chosen for the total sternotomy and the thymus was posteriorly removed without any complications. The pericardium was opened, finding dilation of the right cavities, heparin was administered, and cardiopulmonary bypass (CPB) was initiated with a single venous cannula. The venous cannula was placed in the superior vena cava and the suprahepatic veins that make up the vestige of the inferior cava were clamped.

**Figure 5:**
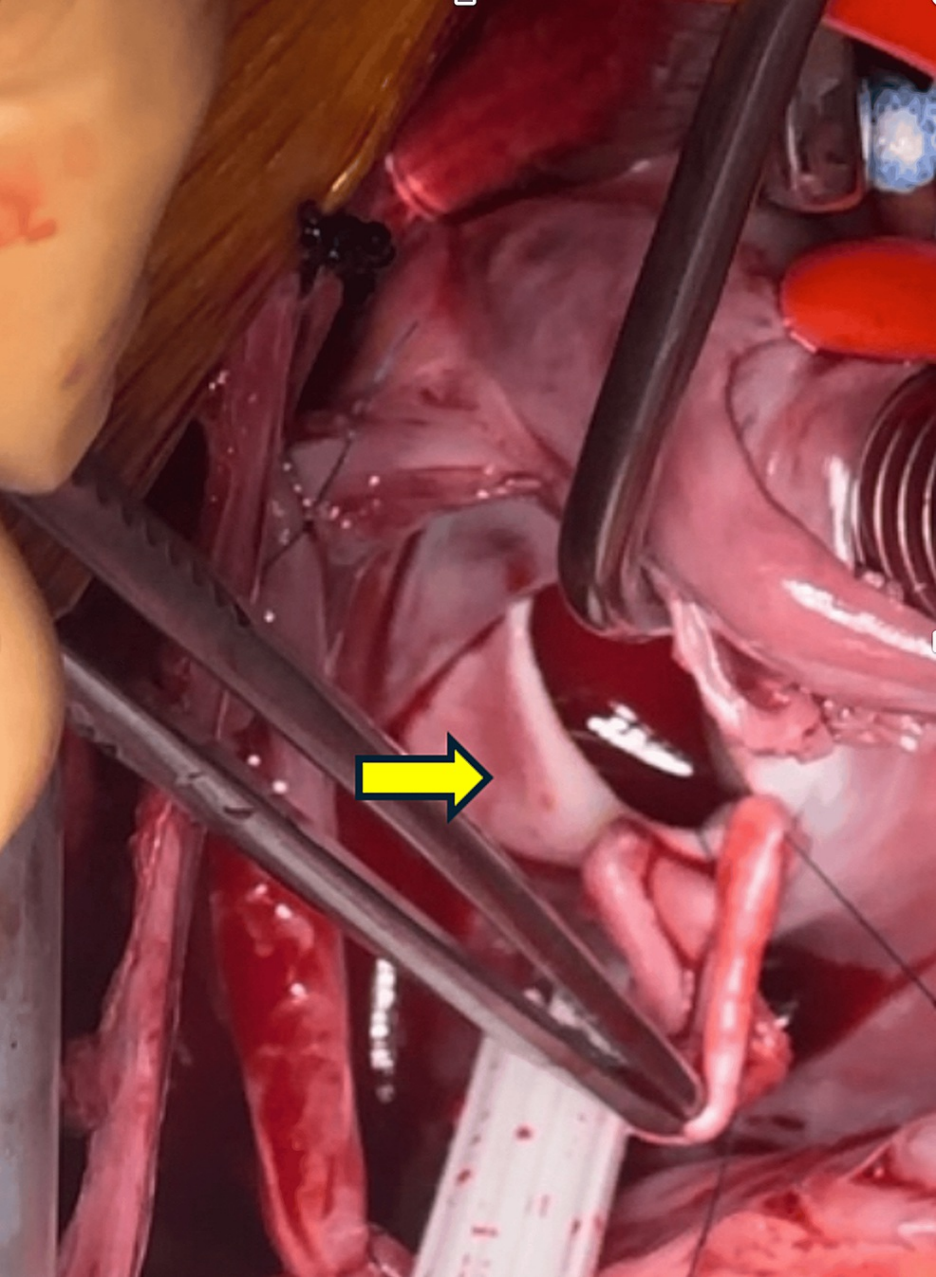
Image of ASD in the patient's heart Interatrial defect of 2.5 x 2 cm with the presence of a forceps angle in the middle defect (yellow arrow) ASD: atrial septal defect

**Figure 6:**
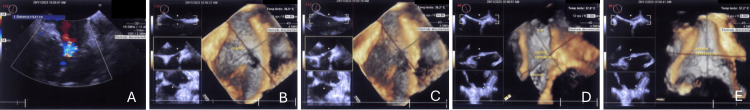
Transesophageal echocardiogram in the operating room A: Contrasted echocardiogram is performed with agitated saline, without evidence of a residual defect in the septum or short circuits due to this approach. B-E: Appropriate placement of the patch, without evidence of residual short circuits. Pulmonary veins are observed without obstruction and the superior vena cava is permeable. The systolic and diastolic function of the left ventricle, without deterioration and without observing complications, associated with the procedure.

The patient was cooled to relative hypothermia, the aorta was clamped and cardioplegia was initiated. The right atrium was opened, finding the defect of 2.5 x 2 cm, which was closed with a pericardial patch (Figure 5). The cardiac cavities were purged, the right auriculectomy was closed posteriorly, and the aorta was unclamped, initiating spontaneous heartbeating. The patient was then rewarmed, and we exited the CPB with decannulation. Epicardial pacing electrodes and drains were placed in the mediastinum and left pleura. Total aortic clamping was 37 min, and cellular recovery hemodiafiltration was used during surgery. The postoperative transthoracic echocardiogram evidenced a tracking of the interatrial septum, observing the adequate placement of the surgical patch without evidence of residual short circuits. A contrast echocardiogram with agitated saline solution is performed, revealing no evidence of a residual defect in the atrial septum (Figure 6).

The postoperative recovery of the patient was without any complication, with a total of five days of hospital stay. Drains in the mediastinum and left pleura were removed on the third postoperative day.

We performed outpatient monitoring for the first and third months without any complications during the physical exploration and echocardiogram imaging.

## Discussion

Sinus venosus type ASD is an uncommon heart abnormality that typically occurs at the entrance of the superior vena cava (SVC) into the right atrium (RA). It is commonly accompanied by abnormal draining of the right pulmonary veins into the RA. The presence of an IVC-type sinus venosus atrial septal defect with an overriding inferior vena cava is an exceptionally uncommon occurrence. The categorization of ASD is determined by the anatomical position within the interatrial septum, the process of embryogenesis, and the size [5].

Inferior SVDs are rare imperfections located in the inferior portion of the atrial septum near the entrance of the IVC. These defects result from abnormal development of the inferior sinus venosus. Abnormal development of this portion of the atrial septum results in an overriding IVC that communicates with both the right and left atria and has the potential for abnormal pulmonary venous return [6].

In our case, the patient had an atrial septal defect of OSD with agenesis of the inferior vena cava being a particularly difficult case to manage via endovascularly. The association between the type of atrial defect of our patient with the vascular variant of agenesis of the IVC is rare compared to the associations with SVDs.

Peacock and Waggstaffe provided descriptions of the pathophysiology of SVD in 1858 and 1868, respectively. In 1956, Ross provided the initial description of the sinus venosus defect, characterizing it as the partial failure of the sinus venosus to merge completely with the right atrium. However, it should be noted that this anomaly is not considered a genuine atrial septal defect [7-9]. The most frequent initial symptom that presents itself is exercise intolerance, which manifests as exertional dyspnea or weariness. Untreated, this illness can advance to Eisenmenger's syndrome and cardiac failure. Untreated ASD has a death rate of 25%. Current guidelines advise that all patients with hemodynamically significant ASD should have ASD closure, irrespective of symptoms, in order to prevent long-term consequences such as atrial arrhythmias, pulmonary hypertension, and/or paradoxical embolism [10-12].

The transcatheter closure is a less invasive approach compared to open surgery being the treatment of choice in the majority of cases. This approach is not indicated in the case of a complex ASD. A complex atrial septal defect (ASD) is characterized by defects larger than 38 mm in diameter and/or defective rims other than the anterosuperior rim. These types of defects are typically not suitable for closure using transcatheter methods and are instead recommended for surgical intervention. Device closure may be contraindicated in cases, where there are additional factors such as young age or low weight, multiple abnormalities, comorbidities, and concomitant heart anomalies [4,13].

The patient in our case had an interatrial size defect of 1.5 cm x 1.2 cm, with a posterior edge of 1.6 cm, upper edge of 0.6 cm, inferior edge of 1.1 cm, and short aortic edge of 4 mm. For the dimensions of the atrial defect, an open surgical procedure was indicated instead of the transcatheter closure.

If the transcatheter closure is not recommended due to certain factors, the alternative treatment option is open surgery. The mortality rate for this procedure is approximately 1% in patients who do not have any serious underlying health conditions. Additionally, less than 7% of patients experience substantial complications following the surgery. Postoperative arrhythmia is the most frequently occurring complication. Surgical closure is the recommended treatment for sinus venosus, primum, coronary sinus defect, and secundum atrial septal defects (ASDs) that cannot be closed using transcatheter methods. The indications for ASD closure are the presence of right heart dilatation without irreversible pulmonary hypertension, as well as the occurrence of paradoxical emboli [2,14].

Currently, the surgery is conducted using a partial mini-sternotomy, which involves making a small incision in the middle of the chest, and a thoracotomy, which can be done either on the right side of the chest or through a vertical incision in the axilla. We conducted a mini-sternotomy in this case to get a more favorable cosmetic outcome [15].

The most favorable result is achieved when the repair is performed before the age of 25. If the operation is done in a younger population, the probability of acquiring late cardiac failure, stroke, and atrial fibrillation is lower in comparison to older patients. Successful closure of a condition results in the reversal of right ventricular remodeling, which is inversely related to the age of the patient. This leads to increased functional class, cardiac output, and exercise capacity, regardless of the patient's age [15].

## Conclusions

ASD secundum is the most common congenital heart defect (CHD). In some cases, the patient may remain asymptomatic in childhood and adolescence with symptoms appearing in adulthood. In the majority of instances, transcatheter closure of secundum ASD has become the preferred method over surgical closure. In scenarios where transcatheter closure is contraindicated, open surgery is the treatment of choice. These patients should be promptly treated to prevent major complications like right ventricular dysfunction and have an adequate postoperative follow-up by a multidisciplinary team.
